# Ambient temperature and subsequent COVID-19 mortality in the OECD countries and individual United States

**DOI:** 10.1038/s41598-021-87803-w

**Published:** 2021-04-22

**Authors:** Costas A. Christophi, Mercedes Sotos-Prieto, Fan-Yun Lan, Mario Delgado-Velandia, Vasilis Efthymiou, Gabriel C. Gaviola, Alexandros Hadjivasilis, Yu-Tien Hsu, Aikaterini Kyprianou, Irene Lidoriki, Chih-Fu Wei, Fernando Rodriguez-Artalejo, Stefanos N. Kales

**Affiliations:** 1 Cyprus International Institute for Environmental and Public Health, Cyprus University of Technology, 30 Archbishop Kyprianou Str., 3036 Lemesos, Cyprus; 2 Department of Environmental Health, Harvard T.H. Chan School of Public Health, Boston, MA USA; 3 Department of Preventive Medicine and Public Health, School of Medicine, Universidad Autónoma de Madrid, IdiPaz (Instituto de Investigación Sanitaria Hospital Universitario La Paz), and CIBERESP (CIBER of Epidemiology and Public Health), Madrid, Spain; 4 Department of Occupational and Environmental Medicine, National Cheng Kung University Hospital, College of Medicine, National Cheng Kung University, Tainan, Taiwan; 5 Department of Medicine, National and Kapodistrian University of Athens, Athens, Greece; 6 Department of Social and Behavioral Sciences, Harvard T.H. Chan School of Public Health, Boston, MA USA; 7 First Department of Surgery, National and Kapodistrian University of Athens, Laikon General Hospital, Athens, Greece; 8 IMDEA-Food Institute, CEI UAM+CSIC, Madrid, Spain; 9 Department of Occupational Medicine, Cambridge Health Alliance, Harvard Medical School, Macht Building 427, 1493 Cambridge Street, Cambridge, MA 02139 USA

**Keywords:** Climate sciences, Diseases

## Abstract

Epidemiological studies have yielded conflicting results regarding climate and incident SARS-CoV-2 infection, and seasonality of infection rates is debated. Moreover, few studies have focused on COVD-19 deaths. We studied the association of average ambient temperature with subsequent COVID-19 mortality in the OECD countries and the individual United States (US), while accounting for other important meteorological and non-meteorological co-variates. The exposure of interest was average temperature and other weather conditions, measured at 25 days prior and 25 days after the first reported COVID-19 death was collected in the OECD countries and US states. The outcome of interest was cumulative COVID-19 mortality, assessed for each region at 25, 30, 35, and 40 days after the first reported death. Analyses were performed with negative binomial regression and adjusted for other weather conditions, particulate matter, sociodemographic factors, smoking, obesity, ICU beds, and social distancing. A 1 °C increase in ambient temperature was associated with 6% lower COVID-19 mortality at 30 days following the first reported death (multivariate-adjusted mortality rate ratio: 0.94, 95% CI 0.90, 0.99, p = 0.016). The results were robust for COVID-19 mortality at 25, 35 and 40 days after the first death, as well as other sensitivity analyses. The results provide consistent evidence across various models of an inverse association between higher average temperatures and subsequent COVID-19 mortality rates after accounting for other meteorological variables and predictors of SARS-CoV-2 infection or death. This suggests potentially decreased viral transmission in warmer regions and during the summer season.

## Introduction

The global pandemic of Coronavirus disease 2019 (COVID-19), which is caused by Severe Acute Respiratory Syndrome Coronavirus-2 (SARS-CoV-2), was first identified in Wuhan, China in December 2019. Since then, it has affected over 213 countries and territories around the world, has resulted in more than 300,000 deaths globally (data accessed on May 15)^1^ and continues to spread.

Evidence suggests that respiratory viruses spread by both person-to person transmission and after hand contact with contaminated surfaces followed by facial self-contamination. Many different factors (the viral agent, the host, and the environment)^2^ may be associated with the rate of viral transmission and subsequent disease severity including: contagiousness, seasonality, ambient temperature, ultraviolet index, humidity, wind speed, air pollution, population density, crowded housing, co-morbidities, etc.^2–5^.

Experimental evidence has found that SARS-CoV-2 is more stable at low humidity and lower temperatures, and decays faster at higher relative humidity and temperatures^6^. The virus is even less stable at higher temperatures combined with simulated solar light (half-life, 3 min)^6^. Similarly, animal studies with the influenza virus showed that the transmission was more efficient at 5 °C than at 20 °C^7^. Additionally, like the SARS-CoV (which caused severe acute respiratory syndrome coronavirus, SARS in 2002), SARS-CoV-2 emerged during the winter months of the Northern hemisphere. However, possible seasonality for SARS-CoV-2 is still debated.

In addition to the potential direct effect of climate on viral survival/infective capacity, the seasonality of respiratory infections may also relate to socio-behavioral changes observed during warmer weather, such as more time spent outdoors and less time spent indoors in more crowded and less ventilated spaces^3^. Casual observation of crude data supported initially lower COVID-19 mortality rates in typically warmer US states (e.g., California, Texas and Florida) and warmer countries (e.g., Australia, Greece, Cyprus, and Israel)^1^.

Recent epidemiological data has suggested that a 1 °C increase in temperature was associated with a statistically significant 3% reduction in daily COVID-19 cases, while a 1% increase in relative humidity was associated with a 0.5% decrease in daily new COVID-19 deaths in 166 countries^8^. Likewise, Liu et al. showed that 1 °C increase in ambient temperature and diurnal temperature range was associated with a decline in daily confirmed case counts in 17 cities in China^9^. In Brazil, dose–response curves suggested a negative linear relationship between higher temperatures and daily cumulative confirmed cases within the range of 16.8–27.4 °C^10^. However, other studies showed no significant association or mixed results between ambient temperature or UV and transmissibility in China^11–14^, incidence and cumulative cases in Spain^15,16^, or epidemic growth^17^. In addition to meteorological conditions, there is research showing geographic differences and population-level factors to be associated with COVID-19 incidence and mortality. These factors include population density, age distribution, and diagnostic testing capacity^18^. Population-based community mitigation measures, such as social distancing, containment, and community-wide mask wearing have also shown to be effective in reducing viral transmission^17,19–21^. Moreover, accumulating evidence demonstrates possible correlations between air pollution and COVID-19 incidence or mortality^22,23^, which therefore, is taken into account in the present study.

As COVID-19 continues to spread and warmer temperatures are expected in most of the Northern hemisphere for the summer, the possible effect of meteorological conditions is emerging as a key question. In light of the preliminary experimental and epidemiologic data, but the lack of definitive evidence regarding mortality, we conducted the current study to provide additional evidence on the associations between ambient factors and COVID-19, using a more hard endpoint, disease mortality, to decrease outcome misclassification and screening bias (incident cases) to differential testing rates. Accordingly, we examined the association of average ambient temperature prior to and after the first reported COVID-19 death, with subsequent COVID-19 mortality rates in the 37 Organization for Economic Co-operation and Development (OECD) countries, the 50 United States (US) and District of Columbia while adjusting for other important meteorological and non-meteorological variables. The OECD is an intergovernmental economic organization founded in 1961, aiming to facilitate world trade, endorse democracy, and commit to market economy. In general, the members of the OECD are regarded as developed countries, sharing similar political values, socioeconomic development and stability, as well as policy responses toward the current COVID-19 pandemic^24^.

## Methods

Data was collected on all US States and the District of Columbia (DC) as well as the other 35 OECD countries from publicly available sources (see supplementary methods). Colombia joined OECD on April 28, 2020 after the start of the pandemic, and therefore, was not included in the current analysis (Table 1). We chose the OECD countries for our study because they provide a group of nations/regions with varied climatic conditions, all of which have been affected by the pandemic. Additionally, to achieve OECD membership, all countries have met certain developmental criteria regarding their economies, infrastructure and political systems, such that they are likely to provide reliable and comparable COVID-19 mortality and covariate data. Data included information on COVID-19 mortality as well as other characteristics, such as average temperature, humidity, and precipitation, air pollution, measures of social distancing, measures of population density, economic and health indices. These are described in further detail below and in Supplementary Fig. S1. In order to adjust for the onset of the epidemic in each region, we used the date of the first local COVID-19 death to define a baseline date for each location from which to collect subsequent mortality outcomes, days of social distancing/confinement metrics, and meteorological data before and after the index date.

**Table 1:** List of the 86 geographic areas included in the study.

Individual states in the US (plus the District of Columbia) (n = 51)	Alabama, Alaska, Arizona, Arkansas, California, Colorado, Connecticut, Delaware, District of Columbia, Florida, Georgia, Hawaii, Idaho, Illinois, Indiana, Iowa, Kansas, Kentucky, Louisiana, Maine, Maryland, Massachusetts, Michigan, Minnesota, Mississippi, Missouri, Montana, Nebraska, Nevada, New Hampshire, New Jersey, New Mexico, New York, North Carolina, North Dakota, Ohio, Oklahoma, Oregon, Pennsylvania, Rhode Island, South Carolina, South Dakota, Tennessee, Texas, Utah, Vermont, Virginia, Washington, West Virginia, Wisconsin, Wyoming
Other OECD countries (n = 35)	Australia, Austria, Belgium, Canada, Chile, Czechia, Denmark, Estonia, Finland, France, Germany, Greece, Hungary, Iceland, Ireland, Israel, Italy, Japan, Latvia, Lithuania, Luxembourg, Mexico, Netherlands, New Zealand, Norway, Poland, Portugal, Slovakia, Slovenia, South Korea, Spain, Sweden, Switzerland, Turkey, United Kingdom

*OECD* The Organization for Economic Co-operation and Development.

### Exposure (average ambient temperature) and meteorological covariates

The primary exposure variable was average ambient temperature over the 25 days prior to the first reported COVID-19 death. The secondary exposure variable was average ambient temperature over the 25 days after the first reported COVID-19 death in order to account for possible differences in the duration of COVID-19 incubation and clinical progression. We also collected relative humidity, and cumulative precipitation, air pollution (average PM_2.5_ levels) data for the same time periods for each region. For data that were available on the city level, we used the first and second largest cities^5,6^ of the individual states and countries. These values were averaged before using in the analysis.

### Non-meteorological covariates

We collected data on the population size, population density, days of social distancing prior to first reported COVID-19 death, the Gini index as a measure of socioeconomic inequality, ICU beds, prevalence of obesity, smoking prevalence, and proportion of the population older than 75 years.

Further details on the sources used for the different variables are provided in the “Supplemental methods information S1”, including the sources for other characteristics that were collected but were eventually not used in the current manuscript. For the exposure and covariates, we retained for analyses only one variable for scenarios where two or more variables were correlated or otherwise not independent (e.g. nursing home residents and persons older than 75 years; PM2.5 and PM10, etc.) and chose the factor with the most complete and reliable information for all regions.

### Outcome variables

The primary outcome of interest was COVID-19 mortality. The data were extracted from open-access databases, namely NY Times^25^ for the US States and DC and Worldometer^26^ for the other OECD countries. We used the date of the first reported local COVID-19 death as the reference date. We then collected the cumulative number of deaths for the period of 25, 30, 35, and 40 days after the first death and computed the corresponding mortality rates per 100,000 population.

### Statistical analysis

Data are presented as mean ± Standard Deviation (SD) for characteristics with symmetric distributions or as median (Quartile 1, Quartile 3) for characteristics with skewed distributions. Deaths at 30 days after the first COVID-19 death was chosen as the main outcome as all regions had accumulated 30 days from the first local death at the time of data collection. Given the over-dispersion in the data, 30-day mortality was modeled using negative binomial regression models with the natural log of the population size used as the offset variable, which has been widely used in other COVID-19 mortality studies^27–29^. The main predictor of interest was the average temperature 25 days prior to the first death and the models were adjusted for a number of other meteorological and non-meteorological covariates, namely average humidity and average precipitation 25 days prior to 1st death, average PM2.5, days of social distancing, density of the largest city, the Gini index, the proportion of the population over 75 years old, the prevalence of obesity and smoking, and ICU beds per million population. In addition to the mortality at 30 days, the 25-day, 35-day, and 40-day mortality were also analyzed. For 35- and 40-day mortality (i.e. secondary outcomes), we used the secondary exposure variable, or the average ambient temperature over the 25 days after the first death (Supplementary Fig. S1 illustrates the lag periods we estimated for viral transmission, disease incubation and clinical progression in fatal cases).

The robustness of the estimates was also checked in other sensitivity analyses, including analyses excluding areas with less than ten deaths over the 30-day period, excluding very cold areas with average temperatures < 0 °C, or excluding areas that could be influential, such as New York, Italy, Spain, Japan, and South Korea for distinct local reasons. In addition, while the NY Times dataset is largely derived from the Johns Hopkins University (JHU) COVID-19 database^30^, which has been widely used for scientific research^31^, we conducted an additional validation analysis using the mortality data directly extracted from the JHU database. The Mortality Rate Ratio (MRR), which is derived by exponentiating the beta coefficient from the negative binomial regression model, together with the corresponding 95% Confidence Interval (CI) and p-value are presented. The Statistical Analysis System SAS version 9.4 (SAS Inc, Cary, NC, USA) was used for all analyses and statistical significance was set at p = 0.05 with all tests performed being two-tailed. The GENMOD procedure in SAS was used for building the negative binomial regression models.

## Results

There was available exposure and outcome information for 86 geographic areas selected for study: including the 50 US States and the District of Columbia as well as the other 35 OECD countries (Table 1). The cumulative number of COVID-19 deaths reported over 30 days after the first local COVID-19 death in each area ranged from 6 (South Dakota) to 9378 (New York).

**Figure 1:**
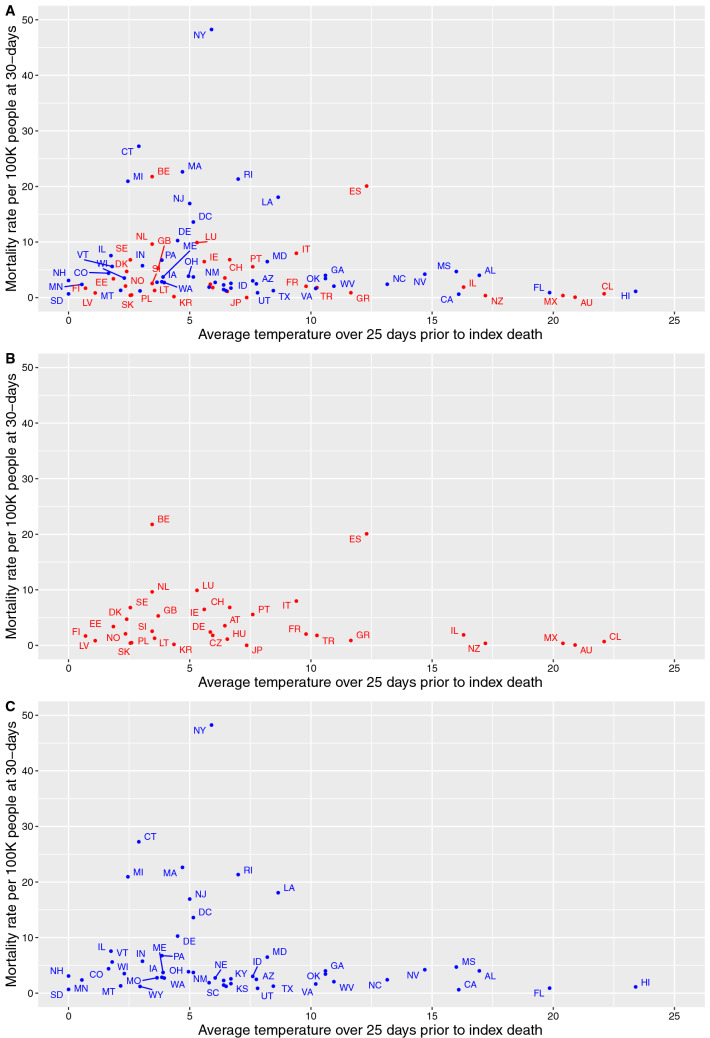
Scatterplot about the 30-day mortality rates (per 100,000 population) and the average ambient temperature over the 25 days prior to the first death in (**A**) all geographic areas together (**B**) OECD countries only and (**C**) in the individual USA states only. *AU* Australia, *AT* Austria, *BE* Belgium, *CA* Canada, *CL* Chile, *CZ* Czechia, *DK* Denmark, *EE* Estonia, *FI* Finland, *FR* France, *DE* Germany, *GR* Greece, *HU* Hungary, *IS* Iceland, *IE* Ireland, *IL* Israel, *IT* Italy, *JP* Japan, *LV* Latvia, *LT* Lithuania, *LU* Luxembourg, *MX* Mexico, *NL* Netherlands, *NZ* New Zealand, *NO* Norway, *PL* Poland, *PT* Portugal, *SK* Slovakia, *SI* Slovenia, *KR* South Korea, *ES* Spain, *SE* Sweden, *CH* Switzerland, *TR* Turkey, *GB* United Kingdom, *AL* Alabama, *AK* Alaska, *AZ* Arizona, *AR* Arkansas, *CA* California, *CO* Colorado, *CT* Connecticut, *DE* Delaware, *DC* District of Columbia, *FL* Florida, *GA* Georgia, *HI* Hawaii, *ID* Idaho, *IL* Illinois, *IN* Indiana, *IA* Iowa, *KS* Kansas, *KY* Kentucky, *LA* Louisiana, *ME* Maine, *MD* Maryland, *MA* Massachusetts, *MI* Michigan, *MN* Minnesota, *MS* Mississippi, *MO* Missouri, *MT* Montana, *NE* Nebraska, *NV* Nevada, *NH* New Hampshire, *NJ* New Jersey, *NM* New Mexico, *NY* New York, *NC* North Carolina, *ND* North Dakota, *OH* Ohio, *OK* Oklahoma, *OR* Oregon, *PA* Pennsylvania, *RI* Rhode Island, *SC* South Carolina, *SD* South Dakota, *TN* Tennessee, *TX* Texas, *UT* Utah, *VT* Vermont, *VA* Virginia, *WA* Washington, *WV* West Virginia, *WI* Wisconsin, *WY* Wyoming.

Summary measures of the major study characteristics considered in the analysis are presented in Table 2. The median mortality rate 30-days after the first reported COVID-19 death was 2.7 per 100,000 population (quartiles: 1.3, 5.6 per 100 K) and the average temperature over the 25-day period preceding the first COVID-19 death had a mean value of 6.7 ± 5.7 °C. Furthermore, the mean number of days of social distancing prior to the first reported death was − 1.0 ± 8.8 days and the proportion of people in the population over the age of 75 years old was 7.4 ± 1.9%. The relationship of the 30-day mortality rates (per 100,000 population) and the average ambient temperature over the 25 days prior to the first death is shown in Fig. 1; Fig. 1A is presenting the scatterplot for all geographic areas together, Fig. 1B for the OECD countries only, and Fig. 1C for the individual USA states only.

**Table 2:** Major study variables for all OECD countries and US states.

Variable	N	Mean	SD	Median	Q1	Q3
COVID-19 Deaths at 30 days after 1st death	86	654	1567	149	52	425
Population	86	15,240,771	25,247,032	5,807,319	2,722,291	11,589,616
COVID-19 Mortality at 30 days after 1st death (per 100 K people)	86	5.35	7.53	2.68	1.29	5.61
Average temperature 25 days prior to 1st death (°C)	86	6.70	5.68	5.70	2.90	8.65
Average PM_2.5_ 25 days prior to 1st death (μg/m^3^)	86	8.15	4.57	7.11	5.46	8.38
Days social distancing before 1st death (days)	86	− 1.03	8.82	− 2.00	− 7.00	5.00
Density of largest city (hundreds of persons/km^2^)	86	29.95	32.61	18.75	9.36	42.30
Gini index	86	0.404	0.084	0.449	0.325	0.472
Population older than 75 years old (%)	86	7.4	1.9	7.1	6.5	8.3
Prevalence of obesity (%)	86	27.9	6.3	28.6	23.6	32.3
Prevalence of smoking (%)	86	18.6	4.7	17.7	15.5	22.0
ICU beds (per million population)	86	199	104	205	97	283
Average humidity 25 days prior to 1st death (%)	86	59.1	19.1	66.4	43.1	72.4
Average precipitation 25 days prior to 1st death (mm)	86	55.7	45.6	44.5	24.6	70.2

35 OECD countries, 50 US states and the District of Columbia.

In multivariable negative binomial regression models (Table 3), the average temperature over 25 days prior to the first death was associated with a statistically significant lower mortality. For a 1 °C increment in average temperature there was a 6% lower mortality rate at 30 days (MRR: 0.94; 95% CI 0.90, 0.99; p = 0.016). Similarly, the number of days enforcing social distancing before the first death was also associated with a lower mortality (MRR: 0.97; 95% CI 0.95, 1.00; p = 0.044). In addition, the density of the largest city (in hundreds of persons/km^2^) and the percentage of the population older than 75 years were associated with increased mortality (MRR: 1.02; 95% CI 1.01, 1.03; p = 0.001 and MRR: 1.21; 95% CI 1.03, 1.41; p = 0.020, respectively).

**Table 3:** Multivariable negative binomial regression model for the mortality at 30 days after the first COVID-19 death.

	MRR (95% CI)	p
Average temperature 25 days prior to 1st death (°C)	0.94 (0.90–0.99)	0.016
Average PM_2.5_ 25 days prior to 1st death (μg/m^3^)	1.01 (0.94–1.08)	0.782
Days social distancing before 1st death (days)	0.97 (0.95–0.99)	0.044
Density of largest city (hundreds of persons/km^2^)	1.02 (1.01–1.03)	0.001
Gini index (per 0.1 increase)	1.36 (0.82–2.26)	0.231
Population older than 75 years old (%)	1.21 (1.03–1.41)	0.020
Prevalence of obesity (%)	1.01 (0.92–1.10)	0.888
Prevalence of smoking (%)	0.96 (0.89–1.03)	0.255
ICU beds (per million population)	1.00 (1.00–1.01)	0.050
Average humidity 25 days prior to 1st death (%)	1.00 (0.99–1.02)	0.726
Average precipitation 25 days prior to 1st death (mm)	1.00 (1.00–1.01)	0.408

*MRR* mortality rate ratio.

In secondary analyses, we assessed the effect of average temperature on COVID-19 mortality in different scenarios to assess the robustness of our results. These scenarios included excluding New York, further excluding Japan and South Korea, excluding areas with less than ten deaths, and excluding areas with average temperature less than 0 °C. The results remained similar when excluding various outliers, including areas with less than ten deaths or with average temperatures less than 0 °C (Table 4). The association of average ambient temperature with lower mortality showed similar effect in stratified analyses within OECD countries and the US States as well (Table 4).

**Table 4:** Average temperature (°C) 25 days prior to first death and mortality at 30 days after the first death.

	MRR (95% CI)^a^	p
**Countries and states**^**b**^		
All (n = 86)	0.94 (0.90–0.99)	0.016
Excluding New York (n = 85)	0.95 (0.90–0.99)	0.026
Excluding New York, Japan, Korea (n = 83)	0.95 (0.91–0.99)	0.011
Excluding areas with < 10 deaths (n = 82)	0.94 (0.89–0.99)	0.013
Excluding areas with temperature < 0 °C (n = 80)	0.93 (0.89–0.98)	0.005
**Countries**		
All countries (n = 35)	0.98 (0.87–1.10)	0.752
Excluding Japan and S. Korea (n = 33)	0.94 (0.87–1.01)	0.093
Excluding Japan, S. Korea, Italy, and Spain (n = 31)	0.92 (0.86–0.98)	0.011
Excluding countries with temperature < 0 °C (n = 33)	0.92 (0.80–1.05)	0.204
**US States**		
All US states (n = 51)^b^	0.94 (0.89–0.99)	0.011
Excluding New York (n = 50)	0.95 (0.90–0.99)	0.036
Excluding states with temperature < 0 °C (n = 47)	0.93 (0.89–0.98)	0.006

*MRR* mortality rate ratio.

^a^Adjusting for average PM2.5 25 days prior to 1st death, days of social distancing before 1st death, density of largest city, Gini index, proportion older than 75 years, prevalence of obesity, prevalence of smoking, ICU beds, average humidity 25 days prior to 1st death, average precipitation 25 days prior to 1st death.

^b^Includes District of Columbia.

We performed other sensitivity analyses. The effect of average temperature on mortality was also statistically significant when the outcome was mortality at 25 days after the first death. Similarly, changing the exposure to the average temperature during the period 25 days after the first death was significantly associated in multivariable models with mortality at 35 days and at 40 days as the outcome (Supplementary Table S1 online). Days of social distancing, density of the largest city, and the proportion of the population over 75 years old also remained significant in these sensitivity analyses.

In the additional validation analysis using the dataset extracted directly from the JHU mortality database, the main findings remain consistent. The multivariate adjusted MRRs for the mortality rate at 30 days were 0.94 (95% CI 0.90, 0.99; p = 0.017) for a 1 °C increment in average temperature, 0.97 (95% CI 0.95, 1.00; p = 0.045) for the number of days enforcing social distancing before the first death, 1.01 (95% CI 1.00, 1.03; p = 0.020) for the density of the largest city (in hundreds of persons/km^2^), and 1.20 (95% CI 1.02, 1.40; p = 0.026) for the percentage of the population older than 75 years, respectively.

## Discussion

In this study we found that after accounting for other meteorological variables and factors predictive of SARS-CoV-2 infection/mortality rates, including age distribution, population density, social distancing, income inequality, and availability of ICU beds, a 1 °C increase in ambient temperature is associated with about 6% lower COVID-19 mortality in the subsequent 30 days, across many countries and the individual United States and District of Columbia. The results were consistent when average temperature was measured during the same and subsequent periods before and after the first reported COVID-19 death and for subsequent mortality at 25, 35 and 40 days; also, they were robust across several sensitivity analyses.

Only a few studies have analyzed the effect of ambient temperature on COVID-19 mortality rates, and to our knowledge none following our approach, thus direct comparison of our results is difficult. Ma et al. found a positive correlation (r = 0.44) between diurnal temperature range and daily deaths, but their analysis is severely limited by the failure to use sufficient time lag periods (only 0–5 days, with non-stable results) and included only the Wuhan population^32^. Wu et al., studied the association between climate and daily new cases and deaths in 166 countries. After adjusting for median wind speed, age, Global Health Security, Human Development Index, and population density, no significant association with temperature and daily deaths was found, although after limiting the analysis to countries with over 10 days since the first reported case and over 100 cumulative cases, a 1 °C increase was associated with a 1.2% reduced number of daily deaths^8^. Again, this study was also limited by short lag periods and by not adjusting for other important prognostic factors, such as social distancing. Finally, Falcao et al. found no association between temperature and COVID-19 deaths but did find an inverse relationship between country average temperature and COVID-19 infections^33^. Our study overcomes the limitations in previous studies by (1) studying mortality rates during different periods after the first reported death in each country or state (25, 30, 35 and 40 days) allowing sufficient time for effects of temperature on viral transmission, incubation and clinical progression using lag times (up to 25–30 days); (2) accounting for other meteorological factors; and (3) including non-meteorological predictors of SARS-CoV-2 transmission and mortality (population density, number of ICU beds, population over 75 years, and social distancing measures to control the epidemic).

Several epidemiological studies have also analyzed the impact of temperature and other weather conditions on COVID-19 incidence, global transmission or reproduction rate with inconclusive results^9,12–17,33–35^. While some studies showed no association between temperature in the range 5–18 °C and cumulative COVID-19 cases such as in Spain^15^, or temperature (median 12.8 °C) and epidemic growth^17^, more recent evidence conducted using worldwide data or data of particular regions including China, Indonesia, Spain, and US are in line with our findings and suggest a beneficial effect of higher temperature on new daily COVID-19 cases^9,34,36–40^. Our results are consistent given that average temperature 25 days prior to the first death was associated with mortality 30 days after, because mortality reflects earlier transmission and clinical disease progression. Specifically, one study has shown that COVID-19 growth rates peaked in temperate regions of the Northern Hemisphere with mean temperature of about 5 °C, and specific humidity of 4–6 g/m^3^, during the outbreak period, while rates were lower both in warmer/wetter and colder/dryer regions^39^, after adjustment for socio-economic variables. Our crude results (Fig. 1) also suggest low mortality in very cold regions, but conclusions are difficult given the small number of observations, the low number of deaths in these areas, and generally the lower population densities of very cold regions. Also in agreement with our results, a recent preprint study has reported an inverse relationship between temperatures above 25 °C and the estimated SARS-CoV-2 reproduction number (3.1% reduction)^34^. Another data-driven study looks into both short-term and long-term COVID-19 transmission patterns, and builds prediction models based on meteorological factors using worldwide data. For the long-term prediction, average temperatures are negatively associated with incident cases two weeks later with a non-linear relationship^41^.

Experimental studies have shown that SARS-CoV-2 can be stable at 4 °C but it is very sensitive to heat: with an incubation temperature of 70 °C the survival time of the virus is 5 min, it achieves over 3 log-unit reduction after 7 days of incubation at 22 °C, and no virus is detected at 14 days; also 1 day of incubation at 37 °C leads to over 3 log-unit reduction in viral level, and no detection afterward^42^. This is in line with evidence on other respiratory viruses, such as SARS-CoV, which shows less stability at 22-25 °C^43^. Moreover, seasonal viruses, like the influenza, peak at winter months with cold weather compromising the host immunity, but also increasing the conditions for person-to-person transmission indoors if ventilation is low and humidity outside is 40–60%^3^. Taking together, this information makes plausible the association of temperature and other weather conditions with SARS-CoV-2 viral infection and death.

Under the assumption that warmer temperatures may help slow down the viral transmission, the summer season may offer an opportunity for public health authorities in each country to strengthen the preparedness and response strategies against a new wave of COVID-19 in the fall. During summer, when in most countries COVID-19 metrics fall, authorities may accelerate vaccination programs to ensure high vaccine coverage before new virus variants, potentially resistant to the existing vaccines, could emerge. Policymakers must also be aware that extreme hot and humid weather may increase social gathering in air-conditioned indoor spaces which, because they are cool and sometimes poorly ventilated, may become ideal environments for virus transmission. Indeed, social distancing measures helped to contain the virus and flatten the peak around the world; in our study, days of social distancing before the first local death was negatively associated with mortality rates at 25–40 days, which is in line with recent research^17^. Finally, unexpected increases in virus transmission or disease mortality, resulting from currently unknown or poorly understood factors, cannot be ruled out. This possibility must also be seriously considered by policymakers and public health officials when enacting or updating COVID-19-related policies.

Of note is that the observed association between ambient temperature and lower COVID-19 mortality holds after accounting for variables, which, as expected, were strong mortality predictors, such as population density, age structure, and economic inequality. There is previous evidence of a greater COVID-19 fatality rates among older people, living either in communities with crowded housing or especially in nursing homes^44^. In our model, we captured these aspects through the Gini index, population density and the proportion of people older than > 75 years^44^. Indeed, there was a significant correlation between the proportion of people over 75 years old and nursing homes (r = 0.46, p < 0.001). Adjustment for population density is also important because it is known that cities with higher density also tend to have more people in crowded housing, higher proportion of socioeconomically disadvantaged persons, and greater income and health disparities, also reflected by the Gini index, which are important drivers of COVID-19 contagion and mortality.

Strengths of our study are: (1) accounting for a good number of meteorological and socio-demographic predictors of COVID-19 infection and mortality; (2) using data on weather conditions before the first reported COVID-19 death, and therefore before most of the countries and US states declared the confinement actions, which could minimize the effect of ambient temperature; (3) analyzing COVID-19 mortality rates, rather than incidence rates, which are less subject to screening and reporting biases, (4) examining the exposure and outcomes across different time periods and in different sensitivity models. Some limitations should also be acknowledged. The most important is that a single measure of ambient temperature may not be fully representative of an entire country or State if it is rather large. Also, we cannot rule out small errors in the dates of the first reported death in each country, due to the different data sources as well as differences in epidemiological surveillance across countries.

## Conclusion

In conclusion, our results showed that a higher average temperature is associated with a subsequent reduction in COVID-19 mortality rates across various developed countries and the individual United States, after accounting for average humidity, precipitation, PM2.5, social distancing, population density, socioeconomic level, the proportion of the elders in the population, the prevalence of obesity and smoking, and ICU beds availability. While the study is limited by possible measurement errors in ambient factors due to using data from the two biggest cities as a proxy, and inherent heterogeneity in the data sources across regions, our main findings remained robust in several sensitivity analyses. Thus, they suggest that mortality from COVID-19 is likely to be reduced during the summer season as a result of warmer weather, possibly decreasing viral survival/infectivity/virulence^45^ and environmental conditions that favor greater time spent outdoors and less indoor gatherings, and thus, less viral transmission.

## Supplementary Information

Supplementary Information.

## Data Availability

Access to the data used can be freely accessed to the scientific community (see “Supplementary data S1”).
