# Supplementary material for: Ambient temperature and subsequent COVID-19 mortality in the OECD countries and individual United States
Source: Sci Rep. 2021 Apr 22;11:8710. doi: 10.1038/s41598-021-87803-w (PMC8062561; doi:10.1038/s41598-021-87803-w)
Supplement: Supplementary file 1 — Supplementary Information. [file 41598_2021_87803_MOESM1_ESM.docx]

**SUPPLEMENTARY MATERIAL**

**Title page**

**Title**: Ambient Temperature and Subsequent COVID-19 Mortality in the OECD Countries and Individual United States

**Authors**: Costas A Christophi, PhD^1,2 †^; Mercedes Sotos Prieto, PhD^2,3, †^; Fan-Yun Lan, MD, PhDc^2,4^; Mario Delgado-Velandia, PhDc^3^; Vasilis Efthymiou, MDc^5^; Gabriel C. Gaviola, MD, MPH^2^; Alexandros Hadjivasilis, MD^1^; Yu-Tien Hsu, MD, MPH^6^; Aikaterini Kyprianou^1^; Irene Lidoriki, PhD^7^; Chih-Fu Wei, MD ^2^; Fernando Rodriguez-Artalejo, MD^3,8^; Stefanos N Kales, MD, MPH^2,9^

^1^Cyprus International Institute for Environmental and Public Health, Cyprus University of Technology, 30 Archbishop Kyprianou Str., 3036 Lemesos, Cyprus. costas.christophi@cut.ac.cy

^2^Department of Environmental Health, Harvard T.H. Chan School of Public Health. Boston, MA, USA.

^3^Department of Preventive Medicine and Public Health. School of Medicine. Universidad Autónoma de Madrid; IdiPaz (Instituto de Investigación Sanitaria Hospital Universitario La Paz); and CIBERESP (CIBER of Epidemiology and Public Health), Madrid, Spain.

^4^ Department of Occupational and Environmental Medicine, National Cheng Kung University Hospital, College of Medicine, National Cheng Kung University, Tainan, Taiwan

^5^Department of Medicine, National and Kapodistrian University of Athens, Athens, Greece

^6^ Department of Social and Behavioral Sciences, Harvard T.H. Chan School of Public Health. Boston, MA, USA

^7^First Department of Surgery, National and Kapodistrian University of Athens, Laikon General Hospital, Athens, Greece

^8^ IMDEA-Food Institute, CEI UAM+CSIC, Madrid, Spain

^9^ Department of Occupational Medicine, Cambridge Health Alliance, Harvard Medical School, Cambridge MA, USA

† **Co-first authors- equal contribution**

**Corresponding Author:**

Stefanos N. Kales MD, MPH,

Occupational Medicine, Cambridge Health Alliance, Macht Building 427

1493 Cambridge Street, Cambridge, MA 02139

Tel. 617/665-1580 Fax. 617/665-1672

E-mail: [skales@hsph.harvard.edu](mailto:skales@hsph.harvard.edu)

**Supplementary Table. Average temperature (°C) and mortality at 25 days, 35 days, and 40 days after the first death**

|  | MRR (95% CI)* | p |
| --- | --- | --- |
| Average temperature over 25 days prior to the first death and 25-day mortality (n=86) | 0.94 (0.90-0.99) | 0.021 |
| Average temperature over 25 days after the first death and 35-day mortality (n=85) | 0.94 (0.90-0.99) | 0.018 |
| Average temperature over 25 days after the first death and 40-day mortality (n=84) | 0.94 (0.89-0.99) | 0.040 |

*Adjusting for average PM2.5 25 days prior to 1st death, days of social distancing before 1st death, density of largest city, Gini index, proportion older than 75 years, prevalence of obesity, prevalence of smoking, ICU beds, average humidity 25 days prior to 1st death, average precipitation 25 days prior to 1st death

**Supplementary Figure 1. Summary of the study design.**

**
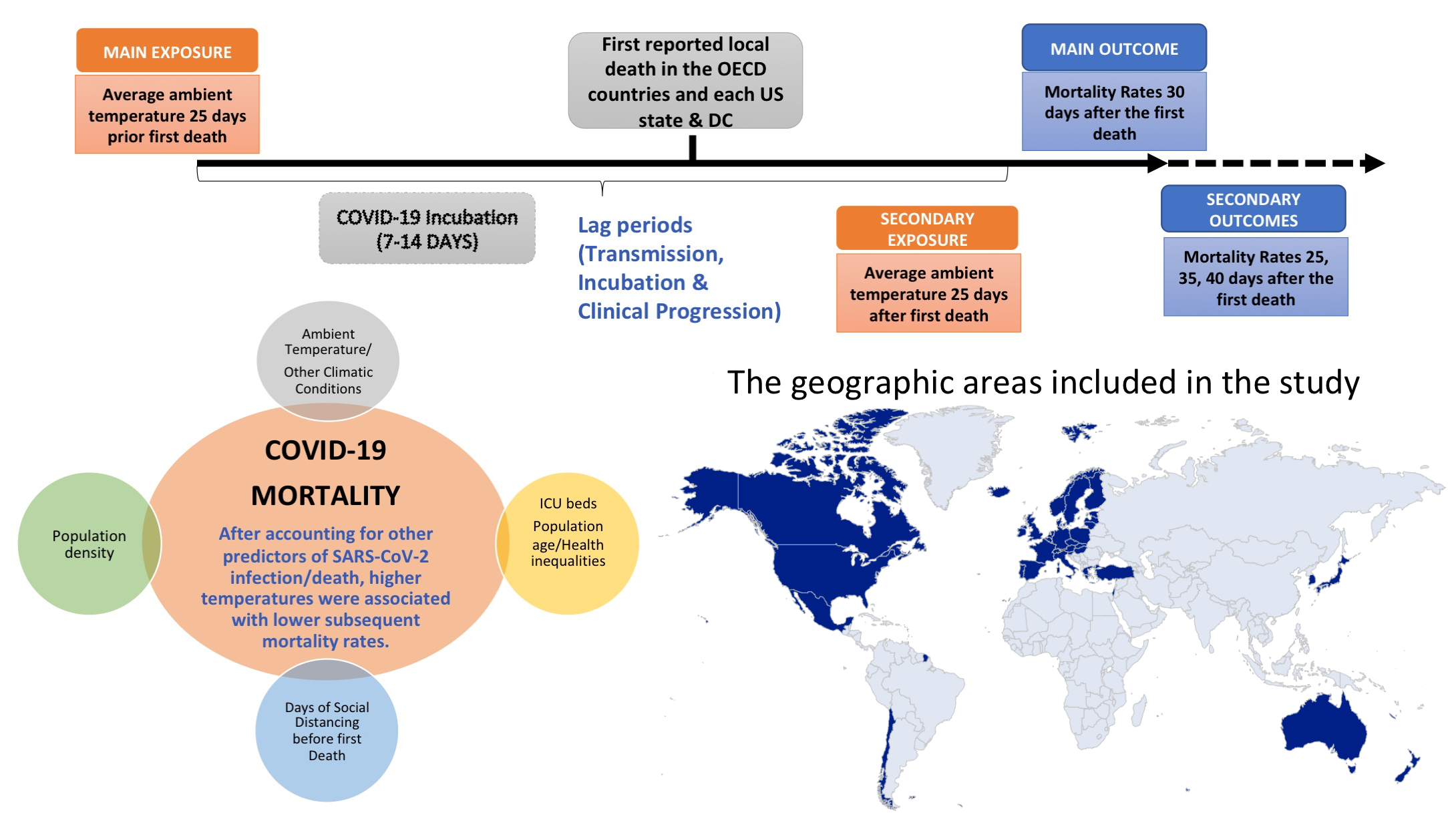
**

The graphic drawing was done using the Microsoft PowerPoint for Mac 2011 (V14.7.7). The map illustrating the geographic areas included in the study was made using the R software (V3.6.3) with “highcharter”, “dplyr”, and “maps” packages.
